# Supplementary material for: The genome sequence of the yellow-legged black legionnaire, Beris chalybata (Forster, 1771)
Source: Wellcome Open Res. 2024 Jun 21;9:151. Originally published 2024 Mar 19. [Version 2] doi: 10.12688/wellcomeopenres.21159.2 (PMC11391196; doi:10.12688/wellcomeopenres.21159.2)
Supplement: Peer review history

# Reviewer report: Reviewer response for version 2

**DOI:** 10.21956/wellcomeopenres.24801.r95939

**Published:** 2024-09-11

**Authors:** Frederick Jaya

**Affiliations:** 1 Sydney Informatics Hub, The University of Sydney (Ringgold ID: 4334), Sydney, New South Wales, Australia

This paper presents a high quality genome assembly of *Beris chalybata*. The paper is well contextualised, outlining the defining characteristics of the species, outlining previous sequencing of closely related taxa, and how this paper fits within the broader scope of the ToL project. 

 The authors generated high coverage PacBio long reads and Hi-C sequencing to generate a complete, high quality assembly following best practices. 

 I appreciate the inclusion of the manual curation outcomes, and the note on uncertainty regarding the X chromosome's orientation - this information will be particularly useful for researchers working with this resource.

 While high coverage RNA-seq data was generated, it is unclear how this data was used. The authors should explicitly describe the role of RNA-seq in the genome annotation. 

 Overall, I have no major concerns with the paper and commend the availability of versioned pipelines, software, and metadata for ensuring reproducibility.

Are sufficient details of methods and materials provided to allow replication by others?

Yes

Is the rationale for creating the dataset(s) clearly described?

Yes

Are the datasets clearly presented in a useable and accessible format?

Yes

Are the protocols appropriate and is the work technically sound?

Yes

Reviewer Expertise:

bioinformatics, comparative genomics, molecular evolution

I confirm that I have read this submission and believe that I have an appropriate level of expertise to confirm that it is of an acceptable scientific standard.

# Reviewer report: Reviewer response for version 2

**DOI:** 10.21956/wellcomeopenres.24801.r87378

**Published:** 2024-07-16

**Authors:** Levente Laczkó

**Affiliations:** 1 University of Debrecen, Debrecen,, Hungary

The authors addressed my comments and now provide a high-quality report on the genome including the analysis process. I have no further concerns about this article.

Are sufficient details of methods and materials provided to allow replication by others?

Yes

Is the rationale for creating the dataset(s) clearly described?

Yes

Are the datasets clearly presented in a useable and accessible format?

Yes

Are the protocols appropriate and is the work technically sound?

Yes

Reviewer Expertise:

genomics, bioinformatics

I confirm that I have read this submission and believe that I have an appropriate level of expertise to confirm that it is of an acceptable scientific standard.

# Reviewer report: Reviewer response for version 1

**DOI:** 10.21956/wellcomeopenres.23404.r80731

**Published:** 2024-05-22

**Authors:** Levente Laczkó

**Affiliations:** 1 University of Debrecen, Debrecen,, Hungary

The authors present the high-quality genome assembly of *Beris chalybata*, in which multiple sequencing approaches were used to reconstruct the complete genome of the species. The background is well described. I agree that genomic resources such as that presented in this article support the in-depth study of species. The sequencing and data analysis methods are appropriate. The primary assembly has high contiguity and the assembly was finalized using Hi-C contact maps, also identifying potential contaminants. The final assembly shows high completeness.

 The Sequencing part has a potential error. I assume that "$HIC_TISSUE" refers to the tissue type used for the experiments, but perhaps it was not interpreted during file conversion(?).

 The methodology is detailed and reproducible, except for the genome annotation. I suggest detailing the gene prediction steps as they could have a large impact on the results. Please also indicate whether soft-masking was used and describe the reference dataset for evidence-based prediction (if it was used). I also suggest using BUSCO in proteome mode and/or OMArk to assess the completeness of the genome annotation.

 The metadata of the samples are very well described, which facilitates the reusability of the data. Apart from what I have described above, I have no concerns about this article.

Are sufficient details of methods and materials provided to allow replication by others?

Yes

Is the rationale for creating the dataset(s) clearly described?

Yes

Are the datasets clearly presented in a useable and accessible format?

Yes

Are the protocols appropriate and is the work technically sound?

Yes

Reviewer Expertise:

genomics, bioinformatics

I confirm that I have read this submission and believe that I have an appropriate level of expertise to confirm that it is of an acceptable scientific standard, however I have significant reservations, as outlined above.
